# A variability in response of osteoclasts to zoledronic acid is mediated by smoking-associated modification in the DNA methylome

**DOI:** 10.1186/s13148-023-01449-1

**Published:** 2023-03-13

**Authors:** Qihua Tan, Anaïs Marie Julie Møller, Chuan Qiu, Jonna Skov Madsen, Hui Shen, Troels Bechmann, Jean-Marie Delaisse, Bjarne Winther Kristensen, Hong-Wen Deng, David Karasik, Kent Søe

**Affiliations:** 1 Epidemiology and Biostatistics, Department of Public Health, University of Southern Denmark, 5000 Odense C, Denmark; 2 Clinical Cell Biology, Pathology Research Unit, Department of Clinical Research, University of Southern Denmark, J. B. Winsløvs Vej 25, 1st Floor, 5000 Odense C, Denmark; 3 Clinical Cell Biology, Department of Regional Health Research, University of Southern Denmark, 7100 Vejle, Denmark; 4 Division of Biomedical Informatics and Genomics, Deming Department of Medicine, Tulane Center of Biomedical Informatics and Genomics, Tulane University, New Orleans, LA 70112 USA; 5 Department of Biochemistry and Immunology, Lillebaelt Hospital, University Hospital of Southern Denmark, 7100 Vejle, Denmark; 6 Department of Regional Health Research, University of Southern Denmark, 5000 Odense C, Denmark; 7 Department of Oncology, Lillebaelt Hospital, University Hospital of Southern Denmark, 7100 Vejle, Denmark; 8 Department of Oncology, Regional Hospital West Jutland, 7400 Herning, Denmark; 9 Department of Pathology, Odense University Hospital, 5000 Odense C, Denmark; 10 Pathology Research Unit, Department of Clinical Research, University of Southern Denmark, 5000 Odense C, Denmark; 11 Azrieli Faculty of Medicine, Bar-Ilan University, 130010 Safed, Israel; 12 Department of Molecular Medicine, University of Southern Denmark, 5000 Odense C, Denmark

**Keywords:** Osteoclasts, Antiresorptives, Zoledronic acid, Epigenetics, DNA methylation, Association studies, Smoking

## Abstract

**Background:**

Clinical trials have shown zoledronic acid as a potent bisphosphonate in preventing bone loss, but with varying potency between patients. Human osteoclasts ex vivo reportedly displayed a variable sensitivity to zoledronic acid > 200-fold, determined by the half-maximal inhibitory concentration (IC50), with cigarette smoking as one of the reported contributors to this variation. To reveal the molecular basis of the smoking-mediated variation on treatment sensitivity, we performed a DNA methylome profiling on whole blood cells from 34 healthy female blood donors. Multiple regression models were fitted to associate DNA methylation with ex vivo determined IC50 values, smoking, and their interaction adjusting for age and cell compositions.

**Results:**

We identified 59 CpGs displaying genome-wide significance (*p* < 1e−08) with a false discovery rate (FDR) < 0.05 for the smoking-dependent association with IC50. Among them, 3 CpGs have *p* < 1e−08 and FDR < 2e−03. By comparing with genome-wide association studies, 15 significant CpGs were locally enriched (within < 50,000 bp) by SNPs associated with bone and body size measures. Furthermore, through a replication analysis using data from a published multi-omics association study on bone mineral density (BMD), we could validate that 29 out of the 59 CpGs were in close vicinity of genomic sites significantly associated with BMD. Gene Ontology (GO) analysis on genes linked to the 59 CpGs displaying smoking-dependent association with IC50, detected 18 significant GO terms including cation:cation antiporter activity, extracellular matrix conferring tensile strength, ligand–gated ion channel activity, etc.

**Conclusions:**

Our results suggest that smoking mediates individual sensitivity to zoledronic acid treatment through epigenetic regulation. Our novel findings could have important clinical implications since DNA methylation analysis may enable personalized zoledronic acid treatment.

**Supplementary Information:**

The online version contains supplementary material available at 10.1186/s13148-023-01449-1.

## Background

Bisphosphonates are used to control and reduce bone loss in a variety of bone pathological conditions, such as cancer-induced bone disease and osteoporosis [1, 2].

Bisphosphonates belong to a class of drugs that are targeted specifically to mineralized bone due to its unique ability, binding to hydroxyapatite. Upon bone resorption, the bone resorbing osteoclast takes up the drug. At this stage, the side-chain of the bisphosphonates will trigger an inhibition of the bone resorptive activity. The N-containing bisphosphonates are reported to target the mevalonate pathway through inhibition of the farnesyl diphosphate synthase (FDPS) (for more details please refer to [1, 2]). Zoledronic acid is a N-containing bisphosphonate and is one of the most potent bisphosphonates to prevent bone loss, as demonstrated in several clinical trials [3, 4]. Despite this, it is also known that it is not equally potent on all patients [5–9]. In the case of cancer patients with bone metastasis around 50% of patients treated with zoledronic acid develop new skeletal-related events despite treatment for 1 year, while with placebo treatment this rate is 70% [5, 6]. Thus, zoledronic acid treatment is not potent on all cancer patients with bone disease. Also for the treatment of osteoporosis, variations in potency of zoledronic acid treatment between individuals are observed [3, 9]. Why may there be such a variation in potency from one person to another? Without a doubt, an answer to this will be multi-factorial, but one priority would be to understand if it is influenced by environmental factors including life-style.

It is well known that the dynamic relationship between bone resorption and formation, which is needed to maintain bone mass and bone health, can be influenced by multiple factors. These include biological factors like age, gender, and menopausal status [10], but also life-style factors like alcohol overuse [11, 12] and tobacco smoking [12–15]. However, it is unclear whether these factors can also affect the potency of drug treatment when using, e.g. zoledronic acid, it seems that insufficient specific knowledge is available regarding this issue [16]. Recent studies suggest that an inadequate response of osteoporosis patients to primarily bisphosphonates correlates with the presence of resistant osteoclasts [17]. This could suggest that an incomplete response of patients to zoledronic acid could be due to a variation in sensitivity of osteoclasts.

We have recently investigated the sensitivity of osteoclasts in vitro to zoledronic acid. These osteoclasts were generated from peripheral blood CD14^+^ monocytes obtained from 46 healthy women. We identified the half-maximal inhibitory concentration (IC50) of zoledronic acid based on total eroded bone surface for each of the osteoclast donors and found a surprising > 200-fold inter-individual variation in sensitivity. Our analyses revealed that smoking was a significant contributor to this variation [18]. Given that smoking is well known to have strong influences on the epigenetic regulation of genes, in particular DNA methylation [19, 20], it is reasonable to speculate that smoking may affect the sensitivity to zoledronic acid through epigenetic regulation. Previous epigenome-wide association studies (EWASs) have demonstrated that cigarette smoking reduces DNA methylation levels at multiple genomic loci in blood cells [21, 22]. Both prenatal and current cigarette smoke exposures have been associated with reduced DNA methylation in genes involved in chemical detoxification such as *CYP1A1* and even hypomethylation at genome level [23, 24]. It is well realized that the environment modulates genetic effects [25], although human genomic studies, e.g. genome-wide association studies (GWASs), and expression quantitative trait loci (eQTL) studies rarely test for genetic interactions with environmental exposures[26].

Epigenetics refer to the meta-level regulation of gene expression caused by mechanisms other than changes in the DNA sequence. Under the constant influence of external factors, epigenetic mechanisms regulate which genes are turned on and off to adapt their expression to a change in environment. As such, epigenetics serves as the bridge between nature, our genome, and nurture, our environment [27]. Different molecular mechanisms are involved in epigenetics, including DNA methylation, histone modifications, and non-coding RNAs (ncRNAs) [28, 29]. In this regard, epigenetic regulation through DNA methylation involves the transfer of a methyl group onto the C5 position of the cytosine to modify the function of the DNA, e.g. to result in gene silencing [27]. In recent studies, we have reported that monocytes, precursors of the human osteoclasts, could have been epigenetically programmed through DNA methylation to reflect age and menopausal status of women, resulting in more aggressive osteoclasts [30, 31]. The significant associations of environmental, including behaviour, factors with bone metabolism call for epigenetic studies to elucidate the molecular mechanisms underlying the interplay between the exogenous factors, nurture, and the genome, nature.

Therefore, we have investigated the smoking-induced epigenetic changes in the genome of peripheral blood mononuclear cells and the possible influence of these epigenetic changes on the sensitivity of resulting osteoclasts to zoledronic acid. We performed an epigenome-wide multifactorial association study on a cohort of pre- and post-menopausal women to investigate the differential DNA methylation regulation patterns associated with the sensitivity to zoledronic acid while taking into consideration the number of cigarettes they have smoked throughout life. Our analyses reveal a strong epigenome-wide association of the DNA methylation patterns at 59 CpG sites with the smoking-dependent sensitivity of osteoclastic bone resorption to zoledronic acid treatment.

## Results

### Characteristics of blood donors

The study group used for the current analyses consists of 34 blood donors out of the 46 used in our previous publication by Møller et al. [18]. The donor characteristics of the original study group can be seen in the original publication [18], while the characteristics of the current sub-group can be seen in Table 1. There are no statistically significant differences between the original samples and the sub-group of samples used for this study with respect to any of the demographic characteristics (mean age 52 vs 53 years—*t*-test *p* = 0.522; median weight 73 vs 72.5 kg—Mann–Whitney test *p* = 0.956; mean height 169.5 vs 169.7 cm—*t*-test *p* = 0.902). In our previous study, we only had information on current smoking status, but after the telephone interview, we can conclude that 12 had never smoked, 16 were past smokers, and 6 were current smokers (Table 1). The median number of cigarettes smoked through a lifetime, including non-smokers, was 6096, but with a large range from 0 to 268,800 (Table 1). The IC50s of zoledronic acid on osteoclastic bone resorption in vitro spanned from 0.061 to 9.49 µM reflecting a 156-fold difference from min to max and with a median of 0.28 µM (Table 1). A comparison of the IC50 values between the original and present study group showed that they did not differ, median 0.26 µM and 0.28 µM, respectively (Mann–Whitney test *p* = 0.8332). Hence, the current sub-group of 34 does not differ from the original study group of 46.

**Table 1:** Demographic characteristics of 34 female blood donors

Patient demographics	
Categories	*n*
*Age*	
40–49	13
50–59	15
60–66	6
*Menopause status*	
Pre-menopausal	15
Post-menopausal	19
*Smoking*	
Never	12
Past	16
Present	6
*Comorbidity*	
No	31
Yes	3
Hypothyroidism	1
Asthma/Allergy	2

Categories	Mean or median (SD^a^) [range]	*n*
Age (years)	52.0 (6.88) [40–66]	34
Height (m)	1.7 (0.05) [1.56–1.80]	34
Weight (Kg)	72.6 (11.8) [55–108]	34
BMI^b^	25.1 (-) [19.49–37.81]	34

Variables	Median [range]	*n*
Cigarettes total	6096 [0–268,800]	34
IC50 in vitro (µM)	0.28 [0.061–9.49]	34

^a^*SD* Standard deviation

^b^Body Mass Index

### EWAS on zoledronic acid IC50 of osteoclast cultures and smoking

**Fig. 1:**
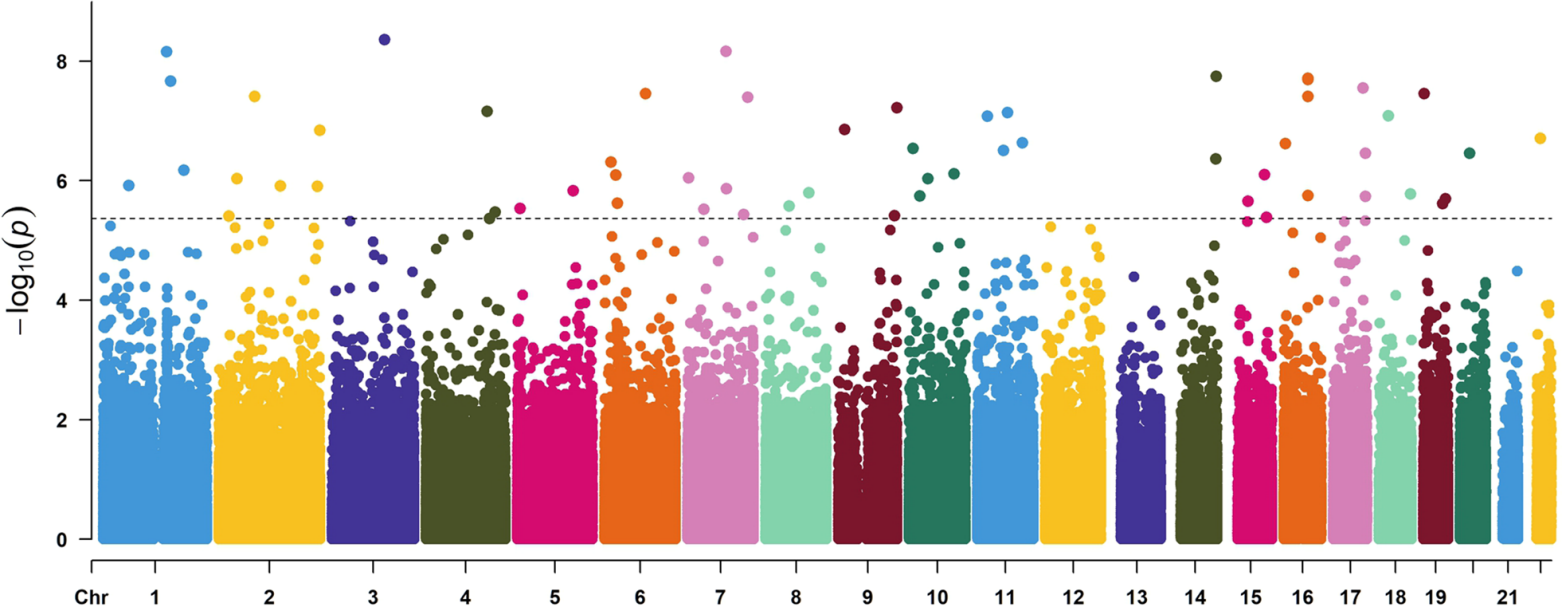
Manhattan plot of the results for smoking-dependent association between CpGs and IC50. *Y*-axis: *p*-value in minus log scale with base 10. *X*-axis: chromosome location by base pairs. The dashed line indicates the *p*-value cut-off corresponding to FDR < 0.05 for genome-wide significance in this EWAS

**Fig. 2:**
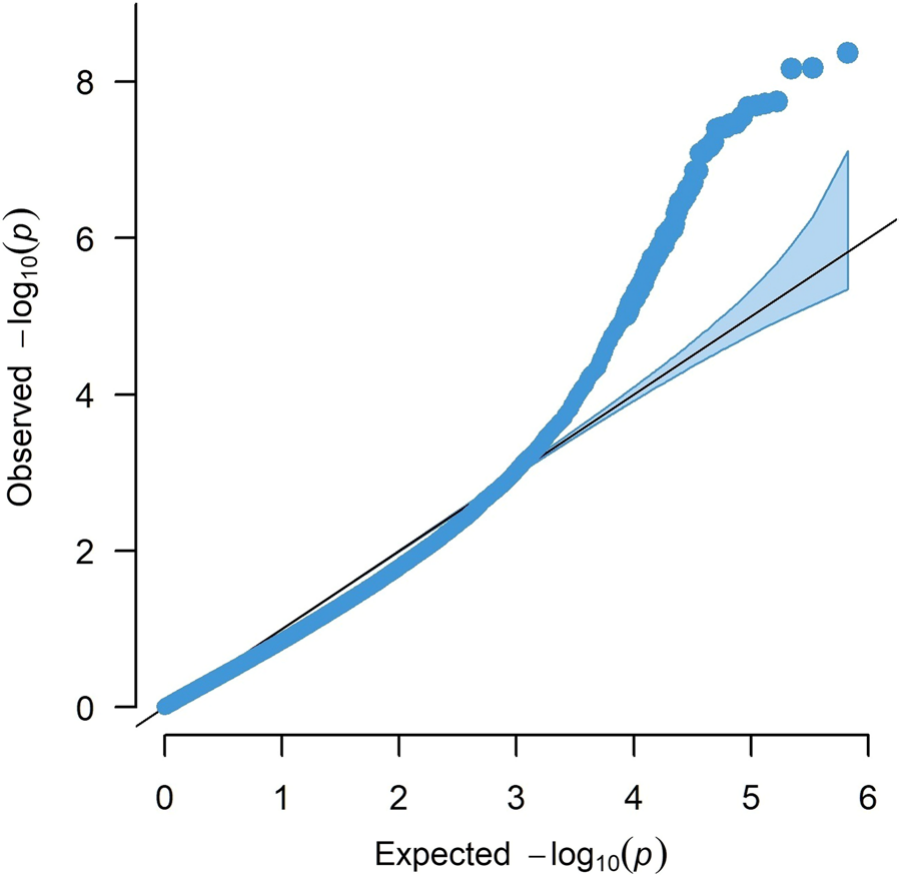
QQ-plot showing the significance levels of the EWAS results for smoking-dependent association between CpGs and IC50. *Y*-axis: observed *p*-value in minus log scale with base 10. *X*-axis: expected *p*-value in minus log scale with base 10

**Fig. 3:**
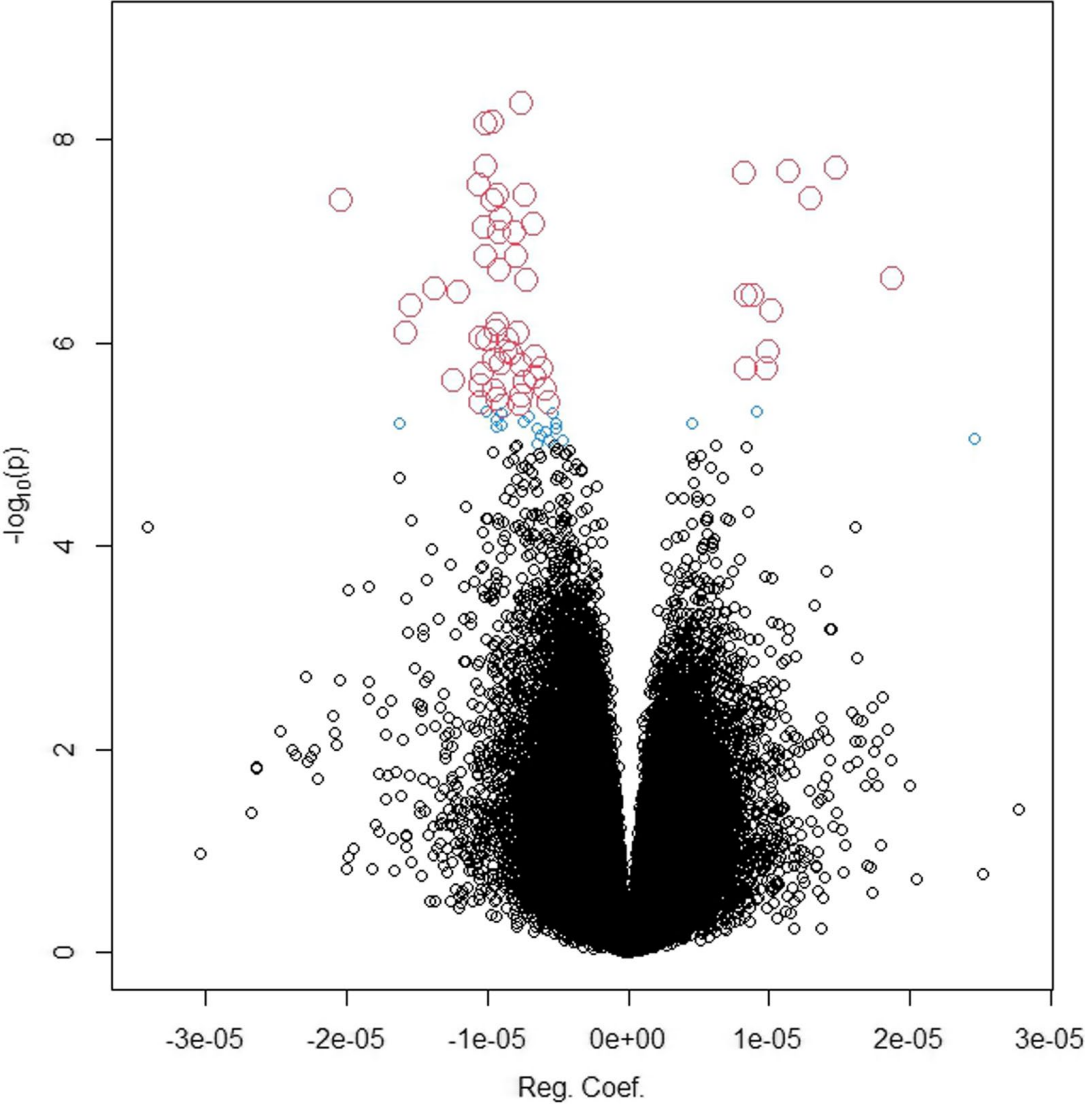
Volcano plot of the smoking-dependent association between CpGs and IC50. *Y*-axis: *p*-value in minus log scale with base 10. *X*-axis: regression coefficient. Color scheme: black circles: not significant; blue circles: suggestive significance; and red circles: genome-wide significance

The EWAS with 865,857 CpG sites was performed using the regression model with DNA methylation as a dependent variable, IC50 and smoking as main effect explanatory variables together with their interaction for assessing the smoking-dependent association between IC50 and DNA methylation. The analysis was corrected for age and cell composition effects. For the smoking-dependent association of IC50 with DNA methylation (i.e., the interaction effect), we identified 59 CpGs displaying genome-wide significance with FDR < 0.05 (corresponding *p*-value < 4.27e−06) (Table 2, Additional file 3: Table S1). A Manhattan plot for the *p*-values and chromosomal location of all the CpGs can be seen in Fig. 1. The QQ plot in Fig. 2 also exhibits a clear pattern of deviation from being random starting roughly from CpGs with *p* < 1e−03. Among the top significant sites, 3 CpGs have *p* < 1–08 and FDR = 2e−03 (cg00227784, cg14355428, cg22010000) (Table 2). The QQ plot in Fig. 2 shows that the top significant sites for smoking-dependent association with IC50 deviate remarkably from the diagonal line of random association. In the volcano plot in Fig. 3, all CpGs reaching genome-wide significance are marked with red color and the CpGs showing suggestive significance are marked as blue. Among the red colored top CpGs, we could see more CpGs with decreased methylation as compared to CpGs with increased methylation.

**Table 2:** The 59 CpGs showing significant smoking-dependent association between IC50 and DNA methylation

Locus	CpG ID	Coefficient	*t*-value	*p*-value	Chr	Position	FDR	Gene	BMD association*
1	cg03009196	9.97E−06	6.35	1.20E−06	1	60,392,511	0.022	CYP2J2	Yes
2	cg22010000	− 1.01E−05	− 8.55	6.89E−09	1	151,738,555	0.002	OAZ3	Yes
3	cg08901901	8.25E−06	8.04	2.14E−08	1	160,951,907	0.002		No
4	cg25619551	− 9.25E−06	− 6.59	6.62E−07	1	193,710,042	0.015		No
5	cg15073625	− 5.60E−06	− 5.88	3.90E−06	2	23,862,014	0.047	KLHL29	Yes
6	cg10565322	− 8.49E−06	− 6.46	9.15E−07	2	43,136,493	0.017		Yes
7	cg17641218	− 9.64E−06	− 7.78	3.87E−08	2	85,804,603	0.002	VAMP8	Yes
8	cg02494004	− 8.62E−06	− 6.35	1.21E−06	2	147,162,808	0.022		Yes
9	cg06342954	− 8.24E−06	− 6.34	1.24E−06	2	236,757,889	0.022	AGAP1	Yes
10	cg14485214	− 7.96E−06	− 7.23	1.42E−07	2	241,957,552	0.005	SNED1	Yes
11	cg00227784	− 7.62E−06	− 8.76	4.33E−09	3	126,391,755	0.002		Yes
12	cg14379327	− 6.77E−06	− 7.54	6.87E−08	4	146,404,134	0.003	SMAD1	Yes
13	cg20093868	− 8.86E−06	− 5.85	4.27E−06	4	152,646,689	0.049	GATB	No
14	cg09865379	− 7.61E−06	− 5.94	3.35E−06	4	165,708,173	0.042	LINC01207	No
15	cg06753918	− 5.91E−06	− 6	2.89E−06	5	6,182,215	0.038		No
16	cg16653901	− 9.45E−06	− 6.27	1.48E−06	5	133,513,511	0.025	SKP1	No
17	cg01247535	1.03E−05	6.72	4.89E−07	6	15,418,354	0.012	JARID2	No
18	cg17866778	− 1.58E−05	− 6.51	8.05E−07	6	26,233,442	0.017		Yes
19	cg06501109	− 1.23E−05	− 6.08	2.37E−06	6	30,850,309	0.033	DDR1	Yes
20	cg17774634	− 9.25E−06	− 7.83	3.45E−08	6	97,730,494	0.002	C6orf167;MIR548H3	No
21	cg05923369	− 1.04E−05	− 6.47	8.93E−07	7	2,251,548	0.017	MAD1L1	No
22	cg24648241	− 9.54E−06	− 5.98	3.01E−06	7	38,347,775	0.039		No
23	cg14355428	− 9.62E−06	− 8.56	6.76E−09	7	91,762,876	0.002	CYP51A1	Yes
24	cg02396891	− 6.61E−06	− 6.3	1.36E−06	7	93,222,734	0.023		No
25	cg10333170	− 9.21E−06	− 5.91	3.66E−06	7	134,521,311	0.046	CALD1	No
26	cg22859658	− 2.03E−05	− 7.77	4.00E−08	7	144,422,735	0.002	TPK1	No
27	cg10993470	− 1.05E−05	− 6.03	2.66E−06	8	55,533,939	0.036	RP1	No
28	cg23855920	− 9.03E−06	− 6.24	1.58E−06	8	103,764,916	0.026		No
29	cg24529650	− 1.02E−05	− 7.24	1.38E−07	9	14,031,418	0.005		No
30	cg13396858	− 1.05E−05	− 5.89	3.85E−06	9	134,249,466	0.047		Yes
31	cg00013660	− 8.96E−06	− 7.6	5.95E−08	9	140,068,770	0.003		Yes
32	cg12368066	− 1.37E−05	− 6.93	2.88E−07	10	9,449,275	0.008		No
33	cg00762372	− 6.16E−06	− 6.19	1.79E−06	10	25,618,736	0.027	GPR158	No
34	cg13820475	− 9.93E−06	− 6.46	9.19E−07	10	44,461,659	0.017	LINC00841	No
35	ch.10.107354959F	− 9.33E−06	− 6.53	7.69E−07	10	107,364,969	0.017		No
36	cg02701677	− 8.07E−06	− 7.46	8.30E−08	11	23,970,707	0.003		No
37	cg08086799	− 1.20E−05	− 6.9	3.12E−07	11	62,495,467	0.009	TTC9C;HNRNPUL2	Yes
38	cg22968863	− 1.03E−05	− 7.51	7.23E−08	11	72,441,739	0.003	ARAP1	No
39	cg20199739	1.88E−05	7.03	2.30E−07	11	107,730,522	0.007	SLC35F2	No
40	cg04932413	− 1.54E−05	− 6.76	4.33E−07	14	103,002,828	0.011	KLC1	Yes
41	cg01279902	− 1.01E−05	− 8.12	1.79E−08	14	104,171,040	0.002	XRCC3	Yes
42	cg04662961	− 6.63E−06	− 6.11	2.21E−06	15	44,486,562	0.031	FRMD5	Yes
43	cg27111704	− 7.75E−06	− 6.52	7.95E−07	15	83,847,628	0.017	HDGFRP3	No
44	cg16362027	− 7.71E−06	− 5.86	4.10E−06	15	88,856,288	0.048		No
45	cg22581270	− 7.20E−06	− 7.01	2.39E−07	16	3,193,156	0.007	CASP16P	Yes
46	cg03660158	9.88E−06	6.19	1.77E−06	16	57,769,342	0.027	KATNB1	Yes
	cg00122310	1.30E−05	7.79	3.84E−08	16	57,769,432	0.002		
	cg00439196	1.48E−05	8.09	1.93E−08	16	57,769,757	0.002		
	cg06830769	1.14E−05	8.07	2.02E−08	16	57,769,885	0.002		
47	cg21854895	− 1.06E−05	− 7.92	2.81E−08	17	71,305,638	0.002	CDC42EP4	Yes
48	cg06400109	8.45E−06	6.18	1.83E−06	17	76,183,118	0.027	AFMID;TK1	Yes
	cg07246050	8.44E−06	6.86	3.44E−07	17	76,183,123	0.009	AFMID;TK1	
49	cg21441526	− 9.09E−06	− 7.46	8.11E−08	18	21,516,439	0.003	LAMA3	No
50	cg26258423	− 7.60E−06	− 6.22	1.67E−06	18	75,380,233	0.027		No
51	cg27071707	− 7.31E−06	− 7.83	3.49E−08	19	1,614,334	0.002	TCF3	Yes
52	cg18788725	− 7.34E−06	− 6.07	2.40E−06	19	45,512,122	0.033	RELB	No
53	cg04700648	− 1.03E−05	− 6.14	2.01E−06	19	52,888,958	0.029	ZNF880	No
54	cg05325193	8.85E−06	6.86	3.47E−07	20	22,558,233	0.009	C20orf56	No
55	cg25580335	− 9.10E−06	− 7.1	1.93E−07	22	24,738,240	0.006	SPECC1L	No

*CpG associated with BMD at *q* < 0.05 within 5 Kb of this locus

**Fig. 4:**
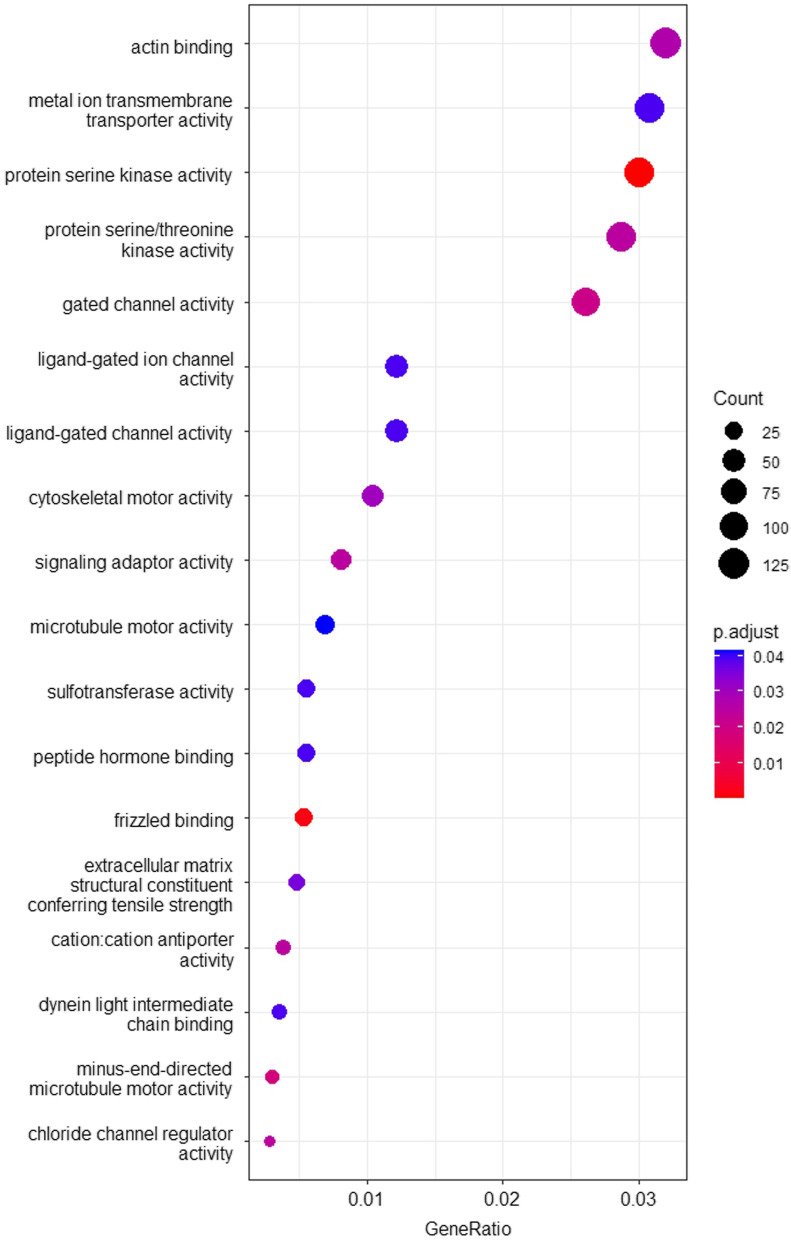
Enriched GO biological processes at FDR < 0.05 for the interaction effect between IC50 and smoking. ORA: over-representation analysis value. Gene Ratio: number of genes in the input list associated with the given GO term

We identified 1 CpG of genome-wide significance for association with the main effect of IC50 (cg00133289, FDR = 0.013, *p* = 1.91e−08) (Additional file 3: Table S1). This CpG relates to the tripartite motif-containing 22 gene (*TRIM22*), coding for a nuclear protein that functions as a nuclear E3 ubiquitin ligase. Functional annotation of differentially methylated CpGs was performed by over-representation analysis of gene ontology (GO) terms using clusterprofiler [32]. Over-representation analysis was performed on 3934 unique genes linked to the 21,760 CpGs associated with the interaction effect between IC50 and smoking with *p* < 0.05. We found 18 GO clusters significantly over-represented by the 3934 genes with FDR < 0.05 (Table 3, Fig. 4). Among the top significant sites are protein serine kinase activity, frizzled binding, minus-end-directed microtubule motor activity, gated channel activity, cation:cation antiporter activity, chloride channel regulator activity, protein serine/threonine kinase activity, signaling adaptor activity, etc.

**Table 3:** Significant GO terms enriched in genes linked to CpGs differentially methylated for smoking-dependent effect of IC50

ID	Description	BgRatio	*p*-value	*p* adjust	*q*-value	Count
GO:0106310	protein serine kinase activity	360/18368	2.99E-07	0.000361978	0.000335646	118
GO:0005109	frizzled binding	37/18368	2.90E-06	0.001755807	0.001628082	21
GO:0008569	minus-end-directed microtubule motor activity	18/18368	4.59E-05	0.018510734	0.017164191	12
GO:0022836	gated channel activity	340/18368	6.92E-05	0.02091644	0.019394897	103
GO:0015491	cation:cation antiporter activity	27/18368	0.000108589	0.024865369	0.023056566	15
GO:0017081	chloride channel regulator activity	17/18368	0.000144847	0.024865369	0.023056566	11
GO:0004674	protein serine/threonine kinase activity	386/18368	0.000150575	0.024865369	0.023056566	113
GO:0035591	signaling adaptor activity	81/18368	0.000164535	0.024865369	0.023056566	32
GO:0003779	actin binding	441/18368	0.000203603	0.027350723	0.025361125	126
GO:0003774	cytoskeletal motor activity	114/18368	0.000250792	0.030320759	0.028115109	41
GO:0030020	extracellular matrix structural constituent conferring tensile strength	41/18368	0.000324922	0.035711918	0.033114094	19
GO:0051959	dynein light intermediate chain binding	27/18368	0.000478613	0.039378783	0.036514217	14
GO:0015276	ligand-gated ion channel activity	143/18368	0.000503203	0.039378783	0.036514217	48
GO:0022834	ligand-gated channel activity	143/18368	0.000503203	0.039378783	0.036514217	48
GO:0046873	metal ion transmembrane transporter activity	430/18368	0.000516627	0.039378783	0.036514217	121
GO:0008146	sulfotransferase activity	52/18368	0.000553713	0.039378783	0.036514217	22
GO:0017046	peptide hormone binding	52/18368	0.000553713	0.039378783	0.036514217	22
GO:0003777	microtubule motor activity	69/18368	0.000617837	0.041498063	0.038479332	27

### Enrichment analyses

Enrichment analyses for the 59 CpG regions showed that at distances ranging from 1,000 to 50,000 bp to the UK Biobank GWAS signals, there was a significant enrichment of 2 genes related to GWAS on bone traits. In addition, 14 genes related to GWAS on body size measures when compared to unrelated GWAS on mental disorders (Table 4; Additional file 1: Figure S1 and Additional file 2: Figure S2).

**Table 4:** Genes enriched for the UK Biobank GWAS signals associated with bone- and body size-related traits

Gene	Max distance (bp)	FDR	*p*-value
GWAS: Bone traits			
* XRCC3*	2500	3E−07	3E−09
* TTC9C*	50,000	3E−04	1E−05
GWAS: Body size traits			
* VAMP8*	1000	3E−05	4E−06
* DDR1*	1000	2E−05	2E−06
* RELB*	1000	3E−02	4E−03
* XRCC3*	1000	3E−02	5E−03
* AGAP1*	2500	8E−05	1E−05
* CYP51A1*	2500	4E−02	9E−03
* ARAP1*	5000	2E−03	3E−04
* KLHL29*	10,000	5E−04	8E−05
* C6orf167;MIR548H3*	25,000	3E−06	3E−07
* HDGFRP3*	25,000	3E−03	8E−04
* JARID2*	25,000	7E−03	2E−03
* LAMA3*	50,000	6E−09	4E−10
* SNED1*	50,000	3E−04	8E−05
* FRMD5*	50,000	1E−03	3E−04

### Independent validation

As an independent validation, we further analyzed the top 59 genome-wide significant CpGs displaying genome-wide significance (*p* < 1e−08) with a FDR < 0.05 for the smoking-dependent association with IC50, in a dataset [33] with multi-omics analyses of BMD in 119 Caucasian female subjects. As indicated in Table 2, among our 59 CpG sites with the smoking-dependent sensitivity to zoledronic acid treatment, 29 were in close vicinity (within 5 kb up- and downstream) of genomic sites significantly associated with BMD (while 30 CpGs had no associations with *q*-value < 0.05 in the dataset [33]). Remarkably, cg04932413 on chr. 14, cg00013660 on chr. 9, cg22581270 in CASP16P, and cg14485214 in SNED1 were highly significantly associated with BMD (*p* < 7.0e−07, FDR < 4.7e−05) (Additional file 4: Table S2). For the main effect of 17 CpGs on IC50 with FDR < 0.1 (Additional file 3: Table S1), 9 CpGs were validated with significant association with BMD (Additional file 5: Table S3).

## Discussion

We have performed EWAS to identify DNA methylation patterns associated with the sensitivity (IC50) of osteoclasts from different individuals to zoledronic acid as well as whether these associations are smoking-dependent. We found 59 CpGs reaching genome-wide significance for the smoking-mediated association with IC50 together with three for the main effects of IC50.

Zoledronic acid, a bisphosphonate, is commonly used to treat osteoporosis and bone metastases. Bisphosphonates target the osteoclast in order to prevent bone resorption [1, 2] and are in general very potent to prevent bone loss, as shown in a multitude of clinical studies [3–6]. However, while the majority of patients have clear benefits of bisphosphonate treatment and seem to be responsive to this treatment, it is not so for all patients. Cairoli et al. [34] conducted a study where 97 patients, who started treatment for osteoporosis with bisphosphonates, alendronate or risedronate, were followed for 3 years. The authors found that 25 out of the 97 patients matched the criteria of treatment failure [9] by meeting at least one of the following criteria: (i) two or more incident fragility fractures and (ii) a decrease in BMD greater than the least significant change (LSC) [35]. In their study, Cairoli et al. [34] found two variables that independently correlated with treatment failure, namely smoking and elevated plasma levels of alkaline phosphatase (ALP) at baseline. Current smoking alone displayed an odds ratio of 3.2, while smoking combined with ALP-levels reached 8.03. This strongly suggests that current smoking increases the risk of treatment failure when using risedronate or alendronate to treat osteoporosis. Only few other studies have addressed the issue and they did not observe any consequence of smoking on the potency of bisphosphonates [16, 36, 37]. In general, these studies primarily focused on the current smoking status or combined it with past smoking. This rough definition of smoking will likely oversimplify something that is very individual, namely the smoking history. Therefore, a strength of our study (though small and conducted ex vivo) is the detailed information on the individual number of cigarettes smoked through life.

In our recent study by Møller et al. [18], current smoking was found to significantly correlate with the sensitivity (IC50) of human osteoclasts prepared from different donors. This phenomenon could suggest smoking as a major contributor to the more than 200-fold variation in sensitivity to zoledronic acid observed between the osteoclasts from different donors [18]. For the current study, new telephone interviews with the participants allowed us to obtain more details on the number of cigarettes smoked through life. This gave us the opportunity to better investigate the potential influence of smoking on the sensitivity of osteoclasts to zoledronic acid.

This updated information allowed us to do EWAS analyses where we investigated if there is a DNA methylation profile of blood cells that may predict the sensitivity (IC50) of osteoclasts to zoledronic acid in the context of cigarettes smoked throughout life. Our analyses indeed identified 59 unique CpGs where the DNA methylation level reflected the number of cigarettes smoked interacting with the IC50 of zoledronic acid. These could be linked to 37 genes. It is important to stress that these 59 CpGs are identified in the DNA based on a blood sample. Since the sensitivity to zoledronic acid is determined in the donor-derived osteoclasts after 10 days of differentiation, it means that the methylation status of the identified CpGs is persistent and may have direct consequences for the sensitivity of the resulting osteoclasts.

In order to identify, which cellular processes are represented by the 21,760 CpGs nominally-significant (*p* < 0.05) for the smoking-dependent association with IC50, we analyzed the 3934 genes linked to these CpGs for over-representation of gene ontology (GO) terms. The analysis identified 18 enriched GO biological processes (FDR < 0.05). It is striking that just a few types of cellular processes are enriched: actin and microtubule-related functions, membrane related transporters and channels, etc. These processes match well-known targets or consequences of N-containing bisphosphonates [38–46] suggesting why the functions and pathways listed in Fig. 4 and Table 3 may affect osteoclasts’ sensitivity to zoledronic acid.

To investigate further the possible function of the 59 CpGs identified in the interaction analysis, we performed an enrichment analysis with existing GWAS databases on bone mineral density and body size measures compared to the unrelated phenotypes—mental disorders. We illustrated the impact of distance from a CpG on the enrichment for GWAS-significant loci associated with relevant traits. By doing so, we identified 15 genes that were significantly enriched within a ≤ 50,000 bp distance between the CpG and SNP for either bone mineral density or body size. These genes are likely to relate to the sensitivity of osteoclasts to zoledronic acid.

In our replication study based on the dataset of Qiu et al. [33], we found that 29 out of the 59 significant CpGs were within 5 k bp of CpGs associated with BMD. Of these 29 CpGs, 8 were hyper- and 21 were hypomethylated, suggesting that most of their related genes will be expressed at higher levels and that this would render osteoclasts more resistant to zoledronic acid. Interestingly, nine of the CpGs found to be significantly associated with BMD (Table 2, Additional file 4: Table S2), were also identified in the GWAS enrichment analysis (shown in Table 4). This therefore suggests a more direct link between these nine genes, their predicted expression level based on DNA methylation changes due to smoking, and BMD as well as sensitivity to zoledronic acid. It is generally known that reduced DNA methylation in the promoter region activates gene expression, while in the intragenic region it may rather reduce expression or even modulate alternative splicing [47, 48]. All nine CpGs were found to be demethylated by smoking, suggesting an increased (if located in promoter) or possibly decreased/aberrant (if located in gene body) expression of related genes. The nine CpGs and their connected genes are: cg15073625 (*KLHL29, body*), cg17641218 (*VAMP8, promoter*), cg06342954 (*AGAP1, body*), cg14485214 (*SNED1, body*), cg06501109 (*DDR1, promoter*), cg14355428 (*CYP51A1, body*), cg08086799 (*TTC9C, promoter*), cg01279902 (*XRCC3, body*), and cg04662961 (FRMD5, body).

The aim of our study was to reveal the molecular basis for how DNA methylation changes in peripheral blood mononuclear cells, triggered by smoking, may cause osteoclasts to become less sensitive to zoledronic acid treatment. Based on all of our presented data, the nine listed CpGs may serve as candidate markers for the underlying molecular basis. However, not much is known about these in the context of sensitivity to zoledronic acid or bisphosphonates. Yet, the following may be of special interest: (1) Vesicle-associated membrane protein 8 (*VAMP8*), belongs to the SNARE family facilitating membrane fusion between late endosomes as well as in autophagy and is closely linked to *VAMP7* [49, 50]. Endosomes play a key role in enabling zoledronic acid to be released into the cytosol of the osteoclast and to act on its target [51]. (2) *CYP51A1* has, to our knowledge, not been reported to have a function specifically in bone cells or osteoclasts. *CYP51A1* is known to be involved in the biosynthesis of cholesterol and acts downstream of FDPS in the mevalonate pathway [52]. However, since we are addressing drug-related effects on the mevalonate pathway, which is not specific to osteoclasts, other non-bone related pathways may also come into play. *CYP51A1* is therefore active in the same pathway that is targeted by zoledronic acid and may therefore explain why a CpG, regulating this particular gene, is the second most significant in our EWAS analysis (Table 2). Furthermore, patients with mutations in *CYP51A1*, amongst other things, display bone defects and reduced cholesterol levels [53]. Thus, the significant validations in Additional file 4: Table S2 and Additional file 5: Table S3 provide further evidence that our identified top associated sites are potentially under regulation by meQTLs, with DNA methylation as an important epigenetic mechanism. This may mediate the interaction between environmental exposure (here smoking) and the genome regulation.

We acknowledge that our study has limitations. A major limitation is the number of samples. More samples may have resulted in additional genome wide significant CpGs, in particular for CpGs related to IC50 alone. This would have allowed a more general view on what determines the sensitivity of osteoclasts to zoledronic acid. An additional limitation is that a comparative analysis should be performed for the identified CpGs in terms of gene expression evaluation, something that has recently been initiated. Finally, our study was done using osteoclasts ex vivo and this of course limits the level of interpretation and impact. However, our study also has multiple strengths. We have foremost used primary human cells as opposed to cells from animals or cell lines. The use of osteoclasts ex vivo as the experimental model allows us to identify specific genes that directly or indirectly play a key role in the sensitivity of the target cells to zoledronic acid. This would not have been possible if only using clinical data from patients undergoing treatment. The combination of detailed demographic information, detailed experimental analysis, and epigenetic analysis has allowed us to obtain strong EWAS data showing that smoking epigenetically regulates genes that are linked to the sensitivity of osteoclasts to zoledronic acid.

## Conclusions

We have identified a mechanism that may explain why past and present smoking negatively affects the clinical potency of drugs such as zoledronic acid [34]. Therefore, this study goes beyond merely identifying changes of DNA methylation due to smoking, since we specifically identify those CpGs that are affected by smoking *and* affect the downstream drug sensitivity of osteoclasts. This has not been shown before. It may give us the possibility to employ a DNA methylation profile of peripheral blood mononuclear cells as a clinical tool to determine who may have optimal or limited benefit of treatment with zoledronic acid. This could enable a more personalized approach to treatment of patients with respect to choice of drug, dosing, and duration of treatment. Of course, this would demand clinical testing to validate such an approach; such investigations are currently ongoing. However, a follow-up to this study by experimental settings should also be conducted to inform the functionality of our candidate meQTLs and further confirm causal genes.

## Material and methods

### The study samples

The study sample consists of 34 female blood donors aged between 40 and 66 (Table 1). They were recruited for the voluntary participation as regular blood donors at the Bloodbank of Lillebaelt Hospital, Denmark, based on the ethical approval on the 11th of May 2015 by the Danish Regional Scientific Ethics Committee (S-20150059) and signed informed consent. A questionnaire was used to collect their personal characteristics, medical history, and lifestyle, while a telephone interview was used to obtain detailed information on their past and present history of cigarette smoking. Through this interview, the following information was collected: (1) present smoker, (2) past smoker, (3) smoked for how many years, (4) stopped smoking when, (5) cigarettes smoked per day. Based on this information we could estimate the total number of cigarettes smoked through their lifetime. The 34 participants reflect a sub-fraction of the participants already used for other publications [18, 30, 31]. These 34 reflect the number of participants that we were able to re-contact for the interview and with respect to demographics and experimental out-come they are fully representative of the original 46.

### Blood sample collection

From each donor, the buffy coat from approximately 500 ml of blood was used to isolate CD14^+^ monocytes for generation of osteoclasts. Furthermore, two 4 ml blood samples were collected when the donors were in a fasting state early in the morning 14 days later than their 500 ml blood donation. One sample was used for extraction of DNA from whole blood. The 4 ml blood samples for collecting DNA were stored at -80 °C.

### Generation of osteoclasts

Osteoclasts were generated according to a standard procedure as described in [31]. In brief, CD14^+^ monocytes were isolated, exposed to 25 ng/ml M-CSF (R&D System, Abingdon, UK) for 2 days followed by exposure to 25 ng/ml of both M-CSF and RANKL (R&D System) for another 7 days with renewal of media twice. Cells were cultured in αMEM (Invitrogen, Carlsbad, CA, USA), 10% FBS (Sigma-Aldrich, St. Louis, MO, USA), and 5% CO_2_ at 37 °C in a humidified atmosphere.

### Determination of osteoclast sensitivity to zoledronic acid and their IC50

In order to determine the sensitivity of osteoclasts of individual donors in vitro the differentiated mature osteoclasts were lifted by Accutase (Biowest BW, Nuaillé, France) treatment and reseeded onto bovine cortical bone slices (BoneSlices.com, Jelling, Denmark), pre-coated with different concentrations of zoledronic acid. Data for the present study was obtained from our previously published study and a detailed description of the procedure can therefore be found in [18].

### DNA methylation analysis

DNA methylation analysis was performed according to the Infinium HD Methylation Assay Reference Guide (document # 15,019,519 v07; https://support.illumina.com/downloads/infinium-hd-methylation-reference-guide-15019519.html). Genomic DNA (0.2–1.0 μg) was bisulfite converted using an EZ DNA Methylation-Direct Kit (Zymo Research, Irvine, CA, USA). DNA samples were bisulfite converted by incubation with the CT conversion reagent for 8 min at 98 °C, 3.5 h at 64 °C, followed by 18 h at 4 °C in a thermocycler. The treated DNA was added to a Zymo-Spin IC Column, desulfonated using M-desulfonation buffer, and then eluted from the column in 12 μl of M-elution buffer. Methylation profiling of the bisulfite-treated DNA was performed using Illumina Infinium MethylationEPIC BeadChip (Illumina, San Diego, CA, USA) according to standard protocol. In brief, 4 μl of bisulfite-treated DNA was denatured, neutralized and amplified with an overnight whole-genome amplification reaction. The amplified DNA was then enzymatically fragmented, precipitated and re-suspended in hybridization buffer before being dispensed onto the MethylationEPIC BeadChips for hybridization. After hybridization, the BeadChips were processed through a primer-extension protocol and subsequently stained. Finally, the BeadChips were coated and imaged using Illumina’s HiScan System.

### DNA methylation data pre-processing

The raw methylation data cover 865,857 CpG sites across the genome. The R package minfi [54] was used for data pre-processing including quality control (QC) and normalization. For each CpG site of a sample, a detection p-value was first calculated by comparing the total signal for each probe to the background signal level estimated from the negative control probes. Very small *p*-values are indicative of a reliable signal whilst large *p*-values generally indicate a poor quality signal. We filtered out 2221 CpGs with detection *p*-value > 0.01 in more than 5% of the overall samples (i.e. 2 samples). After QC, a total of 863,686 CpG sites remained. We further removed all CpGs on the Y-chromosome (535 CpGs) and CpGs physically overlapping with SNPs (181,195 CpGs) leaving 683,408 CpGs for subsequent analysis. We kept X-linked CpGs because all our samples are females. QC at sample level was done by plotting the log median intensity in the methylated (M) against that of the unmethylated (U) channels using getQC and plotQC functions in minfi, with no bad quality sample found. Data normalization was performed by the functional normalization [55] implemented in the *minfi* R-package. At each CpG site, DNA methylation level was summarized by calculating a methylation “beta”-value defined by the Illumina’s formula as *β* = *M*/(*M* + *U* + 100). Before statistical analysis, the *β*-values were converted to methylation *M*-values for better statistical properties by logit transformation with *M* = log_2_(*β*/(1-*β*)) [56].

### Controlling cell-type composition

Since the target tissue is whole blood comprising multiple cell types, cellular heterogeneity among samples can be an important confounding factor in epigenetic association analysis due to cell specificity of DNA methylation. To control for cell-type composition effect, we introduced ReFACTor, a reference-free adjustment for cell-type composition based on principal component analysis (PCA) [57]. The algorithm calculates components that are correlated with the cell-type composition of the samples by applying an unsupervised feature selection step followed by PCA. Instead of estimating absolute cell count values, ReFACTor calculates the linear transformations of the cell-type composition as PCA components.

### Independent validation

We validated the top associated CpGs displaying genome-wide significance (*p* < 1e−08) with a FDR < 0.05 for the smoking-dependent association with IC50, using an independent multi-omics study for BMD [33], which contains DNA methylome data from peripheral blood monocytes in 119 Caucasian female subjects. The selected CpG sites identified in this study, which are located in close vicinity (within 5 kb up- and downstream) with CpGs significantly associated with BMD (FDR *q*-value < 0.05) in the validation study were considered as validated.

### Hypergeometric test

We applied the hypergeometric test for over-representation analysis (ORA) to assess if the overlap of identified markers with those from a functional cluster or category (e.g., biological pathway) is significantly different from being random by calculating a probability from the hypergeometric distribution. ORA has also been implemented in a R package for biological pathway analysis, clusterProfiler [32], to test if members of one biological pathway are over-represented in a list of identified genes.

### Enrichment of GWAS associations in vicinity of the CpGs

We tested the proximity between identified significant CpGs and GWAS-reported SNPs within a specified distance (in base pairs). As stated above, CpGs physically overlapping with SNPs (181,195 CpGs) were filtered out. We downloaded UK Biobank GWAS summary statistics made available by the Neale lab (initially made public on August 1, 2018; http://www.nealelab.is/uk-biobank/). Specifically, we downloaded the sex-combined GWAS summary statistic files listed in the https://docs.google.com/spreadsheets/d/1kvPoupSzsSFBNSztMzl04xMoSC3Kcx3CrjVf4yBmESU/edit#gid=227859291. We kept only traits marked as ‘high confidence’, with estimated heritability > 0.01, and *h*^2^_*z* (*Z*-scores for test of *h*^2^ > 0) ≥ 7. After we filtered out SNPs with *p*-value < 5e−6 there was a total of 393 GWAS datasets.

We further selected the GWAS for traits deemed “relevant” and less relevant for bone physiology. The following grouping was tested: Phen1: BMD and related traits (e.g. arthritis, osteoporosis treatments); Phen2: Body size (weight, height, BMI, length of body parts etc.); Phen3: Smoking and related traits; Phen4: Mental disorders as a “negative control”. The number and list of phenotypes in each ‘Phen#’ group is provided in (Additional file 6: Table S4).

For each CpG and for each Phen#, success ratio was calculated as a proportion of #successful SNPs for that CpG to #all SNPs associated with that phenotype group, Phen#. “Successful” means that the distance between that SNP and particular CpG is less than the stated maximal distance. Series of incremental distances from 1000 to 50,000 bp were tested, and then compared across the phenotype groups. The one-tailed Mann–Whitney *U* test was used to compare differences in success ratios between Phen1 and Phen 2 and “negative control” Phen4 (Additional file 7: Table S5).

### Statistical analysis

We applied the linear regression models to detect the association of DNA methylation with IC50 and smoking as main effects and their interaction effect, adjusting for age and cell composition:

$$\begin{aligned} {\text{DNAm}} & = b_{0} + b_{1} {\text{IC50}} + b_{2} {\text{Smoking}} + b_{3} {\text{IC50}}*{\text{Smoking}} \\ & \quad + b_{4} {\text{Age}} + b_{5} {\text{PC}}1 + b_{6} {\text{PC}}2 + b_{7} {\text{PC}}3 \\ \end{aligned}$$

Smoking was defined as the total number of cigarettes smoked life-long; age was defined as age at blood sampling. Considering the limited sample size of the study, we selected the top 3 principal components (PCs) to add as covariates in the regression model to control for cell-type heterogeneity.

To control for multiple testing, we calculated the false discovery rate (FDR) following Benjamini et al. [58] using the R function *p.adjust*. We define *p* < 1e−05 as suggestive significant and FDR < 0.05 as genome-wide significance.

## Supplementary Information

**Additional file 1**. **Supplementary Figure 1**. Histograms of the success ratio distributions in each phenotype grouping for chosen maximal distance. X-axis, log2 of success ratio (proportion, #successful SNPs/#all SNPs): “Success” means that the distance between this SNP and a CpG in the 59 CpGs set is less than stated maximal distance. Series of incremental distances from 50,000 bp to 1000 bp are shown. Vertical axis of the histogram represents the number of SNPs in each log2 of success ratio’s bin. Data shown corresponds to only SNPs associated with: A) phenotype group 1; B) phenotype group 2; C) phenotype group 3; D) phenotype group 4.

**Additional file 2**. **Supplementary Figure 2**. Histograms of the success ratio distributions in each phenotype grouping for chosen maximal distance. X-axis, log2 of success ratio (proportion, #successful SNPs/#all SNPs): “Success” means that the distance between this SNP and a CpG in the 59 CpGs set is less than stated maximal distance. Series of incremental distances from 50,000 bp to 1000 bp are shown. Vertical axis of the histogram represents the number of SNPs in each log2 of success ratio’s bin. Data shown corresponds to SNPs in all other groups excluding: A) phenotype group 1; B) phenotype group 2; C) phenotype group 3; D) phenotype group 4.

**Additional file 3**. **Supplementary Table 1**. Output of EWAS statistical results (p<0.05) arranged in the order from left to the right for the interaction effect, main effect of IC50 and smoking, followed by annotations for each CpGs in the table.

**Additional file 4**. **Supplementary Table 2**. Validation of top 59 significant smoking-dependent CpGs in an independent multi-omics BMD study [33]. Significant q-values are highlighted in bold, while highly significant q-values are also highlighted in red.

**Additional file 5**. **Supplementary Table 3**. Validation of top CpGs showing main effect on IC50 in an multi-omics independent BMD study [33]. Significant q-values are highlighted in bold, while highly significant q-values are also highlighted in red.

**Additional file 6**. **Supplementary Table 4**. The number and list of phenotypes in each phenotype group. Each sheet is labelled according to the corresponding “phen#” group.

**Additional file 7**. **Supplementary Table 5**. This table shows the raw data for each CpG and each Phen#, success ratio was calculated as a proportion of #successful SNPs for that CpG to #all SNPs associated with that phenotype group, Phen#. “Success” means that the distance between location of a SNP and location of the CpG is less than the stated max distance. Series of incremental distances from 50,000 bp to 1,000 bp were tested, and then compared across the phenotype groups. The one-tailed Mann-Whitney U test was used to compare differences in success ratios between Phen1 and Phen 2 and “negative control” Phen4 as shown in Table 4.

## Data Availability

The datasets used and/or analyzed during the current study are available from the corresponding author on reasonable request.
