# Supplementary figures and images for: A variability in response of osteoclasts to zoledronic acid is mediated by smoking-associated modification in the DNA methylome
Source: Clin Epigenetics. 2023 Mar 13;15:42. doi: 10.1186/s13148-023-01449-1 (PMC10012449; doi:10.1186/s13148-023-01449-1)

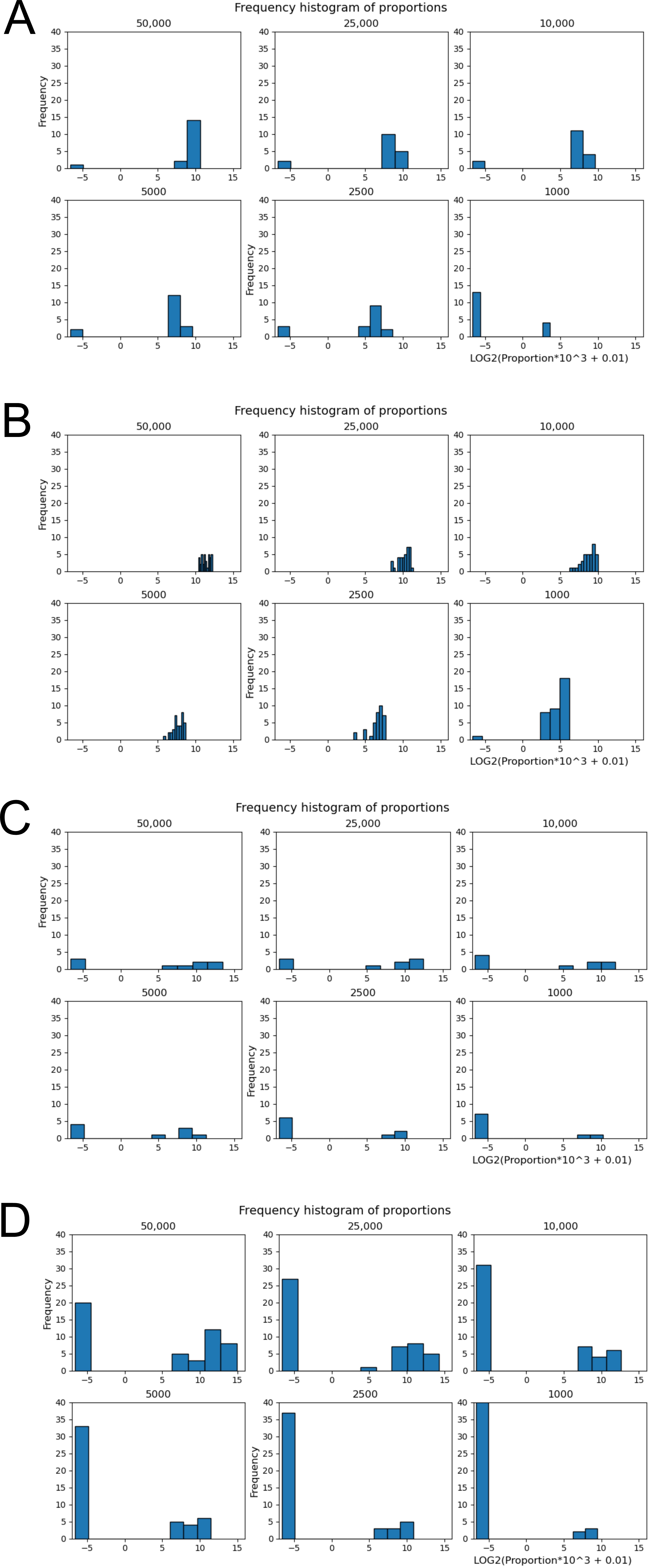

Supplement: Supplementary file 1 — Additional file 1. Supplementary Figure 1. Histograms of the success ratio distributions in each phenotype grouping for chosen maximal distance. X-axis, log2 of success ratio (proportion, #successful SNPs/#all SNPs): “Success” means that the distance between this SNP and a CpG in the 59 CpGs set is less than stated maximal distance. Series of incremental distances from 50,000 bp to 1000 bp are shown. Vertical axis of the histogram represents the number of SNPs in each log2 of success ratio’s bin. Data shown corresponds to only SNPs associated with: A) phenotype group 1; B) phenotype group 2; C) phenotype group 3; D) phenotype group 4. [file 13148_2023_1449_MOESM1_ESM.jpg]

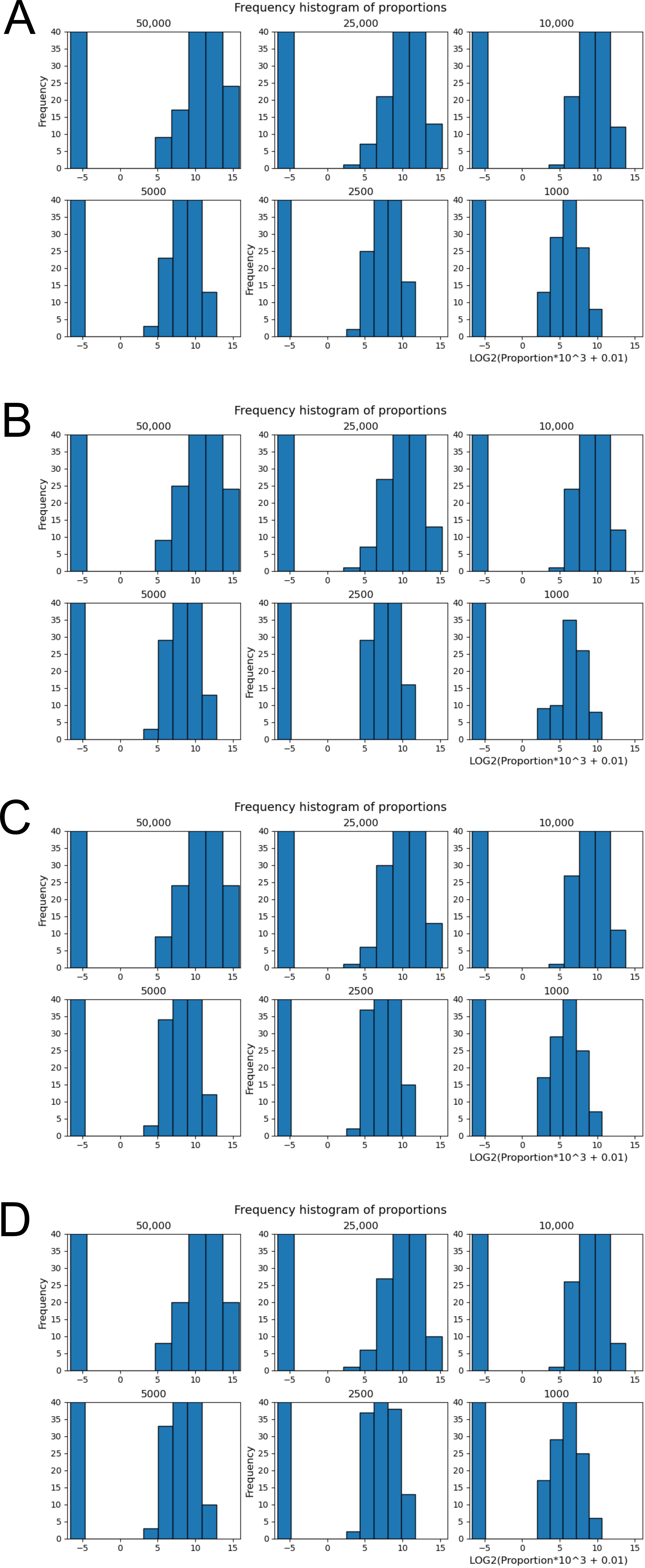

Supplement: Supplementary file 2 — Additional file 2. Supplementary Figure 2. Histograms of the success ratio distributions in each phenotype grouping for chosen maximal distance. X-axis, log2 of success ratio (proportion, #successful SNPs/#all SNPs): “Success” means that the distance between this SNP and a CpG in the 59 CpGs set is less than stated maximal distance. Series of incremental distances from 50,000 bp to 1000 bp are shown. Vertical axis of the histogram represents the number of SNPs in each log2 of success ratio’s bin. Data shown corresponds to SNPs in all other groups excluding: A) phenotype group 1; B) phenotype group 2; C) phenotype group 3; D) phenotype group 4. [file 13148_2023_1449_MOESM2_ESM.jpg]
